# One-sample missing DNA-methylation value imputation

**DOI:** 10.1186/s12859-025-06154-9

**Published:** 2025-05-31

**Authors:** Christelle Kemda Ngueda, Julia Palm, Flavia Remo, André Scherag, Lutz Leistritz

**Affiliations:** Institute of Medical Statistics, Computer and Data Sciences, Jena University Hospital, 07743 Jena, Germany

**Keywords:** DNA-methylation, Missing value imputation, CpG island, Personalized medicine

## Abstract

**Background:**

Currently, the most popular methods for missing DNA-methylation value imputation rely on exploiting methylation patterns across multiple samples from the same population. However, if there is significant variability between individuals or limited data available, these methods might produce biased results. This situation has prompted researchers to seek alternative approaches for handling single-sample data, particularly in the context of personalized medicine. Accordingly, we propose One-Sample Methyl Imputation (OSMI), an imputation method that can also be used in single-sample applications.

**Results:**

The proposed method in single-subject cases yielded an average imputation accuracy of RMSE = 0.2713 (95%-CI from 0.2696 to 0.2730) in β-value units (range: 0–1) based on real 450 K BeadChip data sets of 3,402 individuals. It is possible to take the affiliation of individual CpGs to CpG islands into account during the imputation of missing methylation values. This improves the imputation accuracy. In addition, the accuracy of imputation depends in general on the density of CpG sites on DNA-methylation microarrays and increases as the CpG site density increases. OSMI has low memory and computational requirements.

**Conclusions:**

OSMI uses a single methylome to impute missing values quickly at very low memory constraints. Its imputation accuracy is inferior to other methods if multiple samples are available and these samples are reasonably similar, but OSMI represents a useful addition to the imputation toolbox for the case of single-sample applications.

**Supplementary Information:**

The online version contains supplementary material available at 10.1186/s12859-025-06154-9.

## Introduction

DNA-methylation is an epigenetic process that plays an important role in gene regulation without requiring a change in the DNA sequence itself. This process can be influenced by genetic, environmental, psychological and lifestyle factors, resulting in a unique methylome for each person. As a dynamic and reversible process, DNA-methylation is regarded as a key component in genome regulation [1]. Accordingly, DNA-methylation is of particular interest in personalized medicine, which seeks to customize diagnosis and treatment for individual patients. Currently, thousands of DNA-methylation signals can be quickly measured by either microarray or sequencing protocols in parallel. However, missing values are still a common disadvantage of these technologies, and can significantly impair downstream analyses [2], for instance DNA-methylation clocks [3]. To handle these missing values, several approaches have been proposed from both statistics (e.g. averaging of available measurements, non-parametric probability density estimations, regression-based approaches) and computer science (e.g. iterative soft-thresholding). Specific imputation approaches for missing DNA-methylation data include a linear regression-based approach called methyLImp [4], a regression tree approach called missForest [5] and an approach based on singular value decomposition and weighted k-nearest neighbours called KNNimpute [6]. A key similarity among these methods is that they are applicable to both DNA-methylation microarray and sequencing data, and that they require DNA-methylation data from at least two samples (either from two individuals or from repeated measurements of the same individual). Thus, the methods fundamentally rely on similarities in DNA-methylation between individuals from the same population. However, if this assumption is violated due to large inter-individual variability [7], biased results may be expected [8, 9]. Accordingly, there is a need to develop an imputation technique suitable for single-sample applications, particularly in personalised medicine that takes into account the specific conditions and needs of individual patients rather than small, strictly defined patient groups [10].

We have developed a method called One-Sample Methyl Imputation (OSMI), which is inspired by the observation that DNA-methylation in mammals occurs almost exclusively at sites where a nucleotide carrying the cytosine base is linked to a nucleotide carrying the guanine base by a phosphate group (CpG sites). The closer these CpG sites are to each other within the same sample, the greater the probability that they have the same methylation level [11]. Thus, OSMI replaces missing DNA-methylation values with the closest available value on the chromosome within the strand of a single sample. This approach might enable us to bypass the usual need to account for properties and relationships across data from multiple individuals.

We compared OSMI with other two established methods (methyLImp and impute.knn) using publically available DNA-methylation microarray data from the Epigenome-Wide Association Study (EWAS) data hub.

In addition, we present a variant of OSMI that specifically exploits information about CpG islands structure. CpG islands are genomic regions, mostly located in promoters, characterized by a high frequency of CpG dinucleotides [12]. As CpGs within a CpG island tend to have similar methylation patterns, this (correlation) information may be used for imputation purposes.

## Materials and methods

### Data

This work is based on data from the EWAS data hub which is a subdomain of the open source platform EWAS (Epigenome-Wide Association Study) (https://ngdc.cncb.ac.cn/ewas/datahub/download, version v1.20210621). The data hub provides data sets with minimal individual phenotype information and DNA-methylation array data (Infinium HumanMethylation450 and MethylationEPIC BeadChip) [13]. Here, we focused on the blood methylation data set (named ‘blood_methylation’ on EWAS [14]), which consists of 485,512 CpG sites and 3,402 samples, together with the corresponding phenotype data set containing the chronological age of 1,872 individuals (named ‘sample_blood_methylation’ on EWAS [15]). The blood methylation data set has approximately 10% missing values, with a range of 0.3% to 90.8% missing values per sample and 0% to 99.9% missing values per CpG site.

In addition, we made use of information on CpG start positions, information about chromosomes and strands as well as the affiliation to CpG islands from two data files: http://hgdownload.cse.ucsc.edu/goldenpath/hg38/database (file: cpgIslandExt.txt.gz [16]) and https://bioconductor.org/packages/IlluminaHumanMethylation450kanno.ilmn12.hg19 (file: IlluminaHumanMethylation450kanno.ilmn12.hg19_0.6.1.tar.gz [17]).

### Data pre-processing

The publicly available data sets from the EWAS data hub have already been pre-processed. Signal intensities are trimmed using the Minfi package of Bioconductor and data are normalised using an in-house based method and the Beta-Mixture Quantile Normalisation [18].

**Fig. 1:**
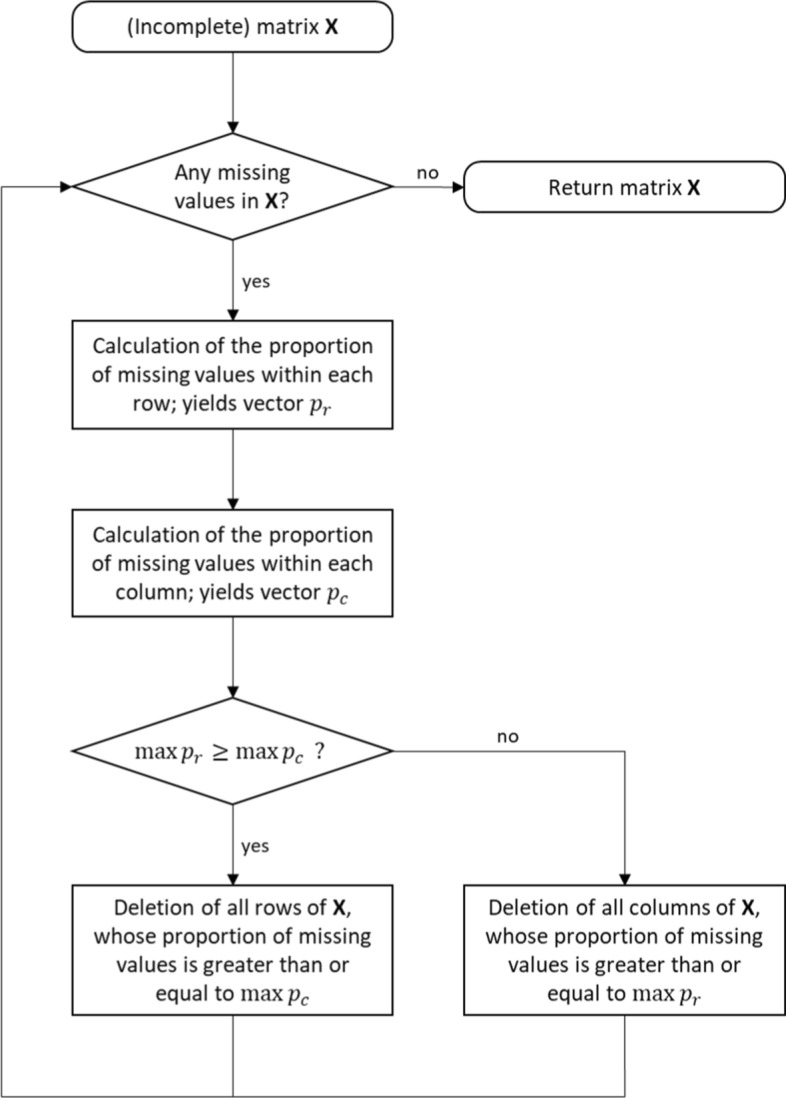
Flowchart of the procedure for reducing raw data containing missing values to a complete data subset without missing values

In addition, we filtered the raw data set, such that the final data set did not contain any missing DNA-methylation values. To do this, we applied a simple and fast greedy algorithm based on the following heuristics: Without loss of generality, let $\mathbf{X}$ be a $(m,n)$-matrix containing missing values with $m\ge n$. Based on $\mathbf{X}$, the percentages ${p}_{r}(k)$ and ${p}_{c}(l)$ of missing values are determined in each row $k$ and column $l$, respectively. If $\text{max}\,{p}_{r}\ge \text{max}\,{p}_{c}$ holds, all rows $k$ of $\mathbf{X}$ with ${p}_{r}\left(k\right)\ge \text{max}\,{p}_{c}$ are deleted from $\mathbf{X}$. Conversely, in the case of $\text{max}\,{p}_{r}<\text{max}\,{p}_{c}$, all columns $l$ of $\mathbf{X}$ with ${p}_{c}\left(l\right)\ge \text{max}\,{p}_{r}$ are deleted. This process is repeated iteratively based on the newly created smaller $\mathbf{X}$ matrix until all missing values have been removed. The procedure must be iterative because deleting rows may change the column percentages ${p}_{c}$ of the remaining matrix and deleting columns might modify the row percentages ${p}_{r}$, respectively. The entire procedure is shown as a flowchart in Fig. 1.

Applied to the original data set of size 485,512 × 3402, this produced a complete data set with 279,613 CpG sites and 2960 samples. Ultimately, it served as ground truth in the evaluation of various imputation methods considered later.

#### Data set I: comparison of imputation methods

**Fig. 2:**
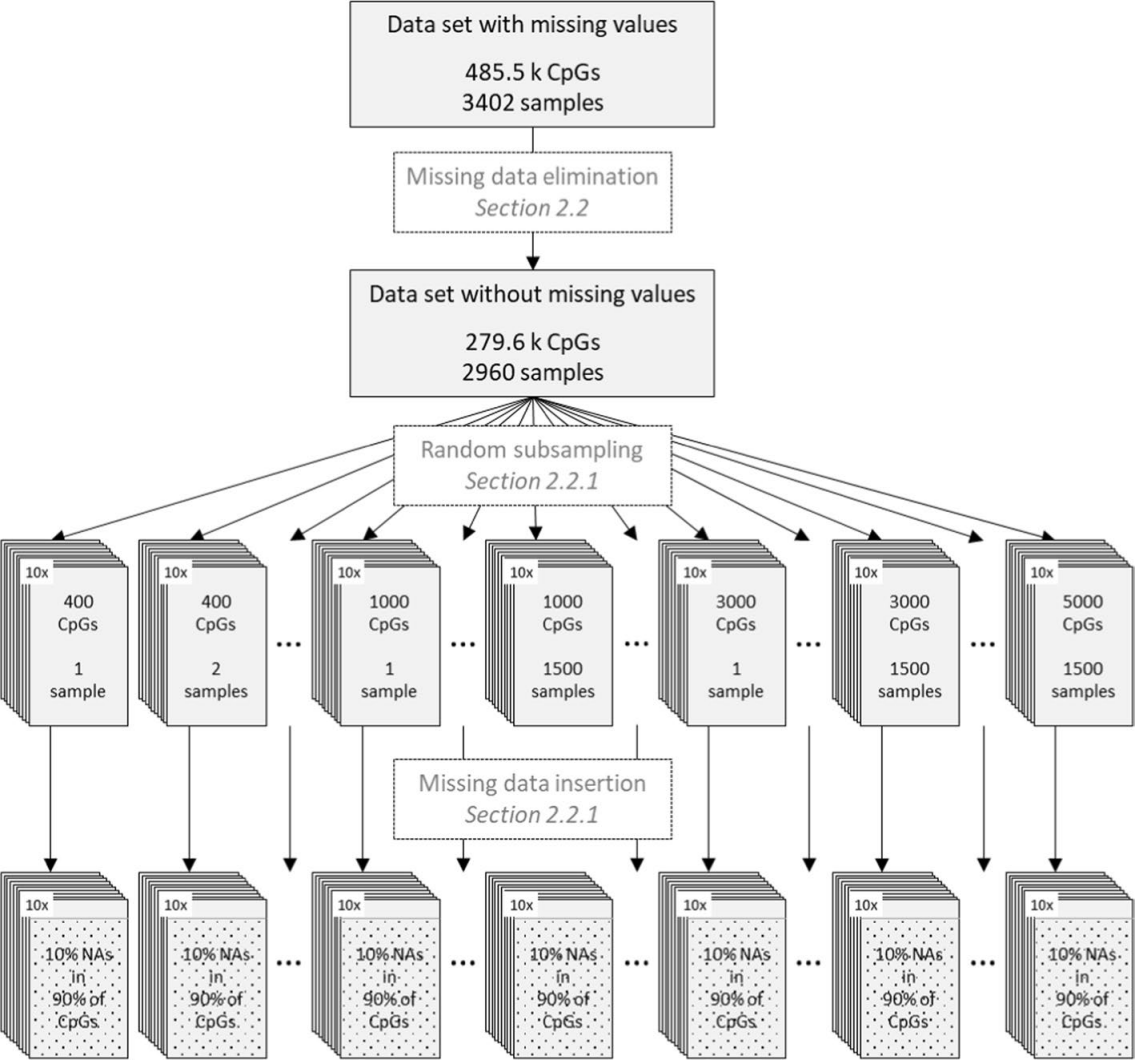
Workflow for generating simulated data sets with missing values and known ground truth

Due to limitations of computational resources, we randomly sampled 10 realizations of 36 smaller subsets of different size combinations each with 1, 2, 5, 10, 50, 100, 500, 1000, or 1500 samples and 400, 1000, 3000, or 5000 CpG sites from the complete cases data set. In each subset, we introduced 10% missing values completely at random in 90% of CpG sites to retain some complete case CpG sites as required by the MethyLImp approach [4]. A schematic workflow from the original data set via a reduced data set without missing values to various simulated data sets with known missing values is illustrated in Fig. 2.

#### Data set II: impact of CpG island structure

To explore the impact of CpG island structure on the imputation accuracy, we compiled a data subset that contains only CpGs that can be assigned to a CpG island and have at least one other CpG site with an existing methylation value within the same island. This resulted in a complete data set with 117,132 CpGs and 2,960 samples (see Table 1). Again, 10% of missing values were introduced completely at random into this data set.

**Table 1:** Summary of the dimensions of all data sets I–IV

Data set	Number of CpGs	Number of samples
I	400/1,000/3,000/5,000	1/2/5/10/50/100/500/1000/1500
II	117,132	2960
III	485,512	3402
III	353	1872

#### Data set III: impact of the density of a methylation array

To investigate the influence of the available CpG sites on the imputation accuracy, we used the original blood data set as the starting point and processed it sample by sample, such that each sample only contained complete CpG records. This resulted in 3,402 samples with different numbers of available CpGs per sample (see Table 1). Subsequently we introduced 10% missing values completely at random into DNA-methylation values of each sample.

#### Data set IV: imputation effects on DNA-methylation clocks

In order to investigate the impact of different imputation strategies for DNA-methylation clocks [19, 20], we used the original blood data set restricted to samples, for which the chronological age was available (485,512 CpGs and 1,872 samples) and, for technical reasons, a second data set further reduced to the CpGs required for Horvath´s Clock (353 CpGs and 1,872 samples, see Table 1) [19, 20]. We focussed on Horvath´s Clock given that results were qualitatively similar for other clocks.

### One-sample methyl imputation method

The OSMI method replaces missing DNA-methylation values with measured values of the closest CpG site within the same microarray. In the method, the distance between two CpG sites is defined as the difference in base pairs between their positions on the same chromosome strand. To achieve this, each CpG of the array must initially be annotated with its corresponding chromosome name, position, and strand.

Below, we describe the OSMI method: For each chromosome and strand, let ${\varvec{\upbeta}}$ and $\mathbf{p}$ be two vectors of the same size, which denote DNA-methylation values and positions of CpG sites, respectively. Both vectors may contain missing values. We call pairs $\left({\beta }_{k}, {p}_{k}\right)$ complete CpG records when neither the DNA-methylation value nor the CpG site position is missing. Depending on particular availabilities of complete CpG records within a chromosome, we must differentiate between two cases.

**Fig. 3:**
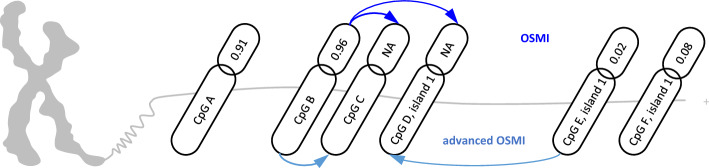
Schematic representation of the replacement of missing DNA-methylation values by measured values of the nearest CpG site within the same sample: without (OSMI) and with (advanced OSMI) the use of CpG island structures

1. Initially, let us assume that there is at least one non-missing value in the vector ${\varvec{\upbeta}}$ available. In case the DNA-methylation value of a CpG site is missing but the position is available, the missing DNA-methylation value is imputed by the value(s) given by the nearest complete CpG record(s). Thereby, the nearest neighbour of a CpG (or the two nearest neighbours, respectively) is defined as the complete CpG record, which has the smallest distance in base pairs to the CpG site under consideration (e.g. the average DNA-methylation values of both nearest neighbours, respectively), see Fig. 3. When the position of a CpG is missing as well, no neighbouring CpG can be determined. In this case, OSMI imputes the missing DNA-methylation value as the mean value of all available DNA-methylation values in ${\varvec{\upbeta}}.$
2. In exceptional cases, restriction to particular chromosomes and strands may result in vectors ${\varvec{\upbeta}}$ that do not contain any measured DNA- methylation value. Obviously, if all DNA-methylation values in ${\varvec{\upbeta}}$ are missing, the previously described algorithm fails. In this case, missing DNA-methylation values are imputed by the genome-wide average of available DNA-methylation values.

By construction, OSMI has low memory requirements, since it only requires two vectors with DNA-methylation values ${\varvec{\upbeta}}$ and the associated CpG site positions $\mathbf{p}$. Generally, this approach has the advantage that all DNA-methylation can be analysed downstream without restrictions. It enables a better exploitation of correlations between nearby CpG sites especially in the case of densely arranged CpG sites.

OSMI can be easily modified, such that similarities in methylation within CpG islands are taken into account. To achieve this, step (1) is modified if a CpG with missing methylation information is located inside a CpG island and there is at least one complete CpG record within the same island. Then the next neighbour search can be restricted within the corresponding island instead of searching on the whole chromosome and strand. In all cases where this modification is not feasible, OSMI is used in its original form instead. We will call that variant advanced OSMI (see Fig. 3).

### Performance measures

To assess the performance of OSMI and other imputation approaches, we consider the root mean square error (RMSE) as well as the mean absolute error (MAE) between ground truth DNA-methylation values and the corresponding imputed values. Similarly, we calculated these errors between predicted and chronological age. In addition, we provide estimates of computational time for various scenarios.

We compared OSMI with methyLImp (version 0.99.4, https://github.com/pdilena/methyLImp, [4]) as well as the weighted k-nearest neighbours’ method impute.knn (from package ‘impute’ version 1.78.0) each with default settings (https://www.bioconductor.org/packages/release/bioc/html/impute.html, Bioconductor—methyLImp2, version 1.0.0). The selection of these two methods was based on the performance, computing time and memory requirement comprehensively evaluated by Di Lena et. al. [4]. All analyses were carried out in R (version 4.1.2) installed on a Linux CentOS, X86_64 system with 2 Intel (R) Xeon (R) Gold 6154 CPUs @ 3.00 GHz processors, 72 CPU and 256 GB of RAM.

## Results

### Comparative considerations on imputation accuracy

The following results were obtained on the basis of data set I (see Sect. “Data set I: Comparison of imputation methods”).

**Fig. 4:**
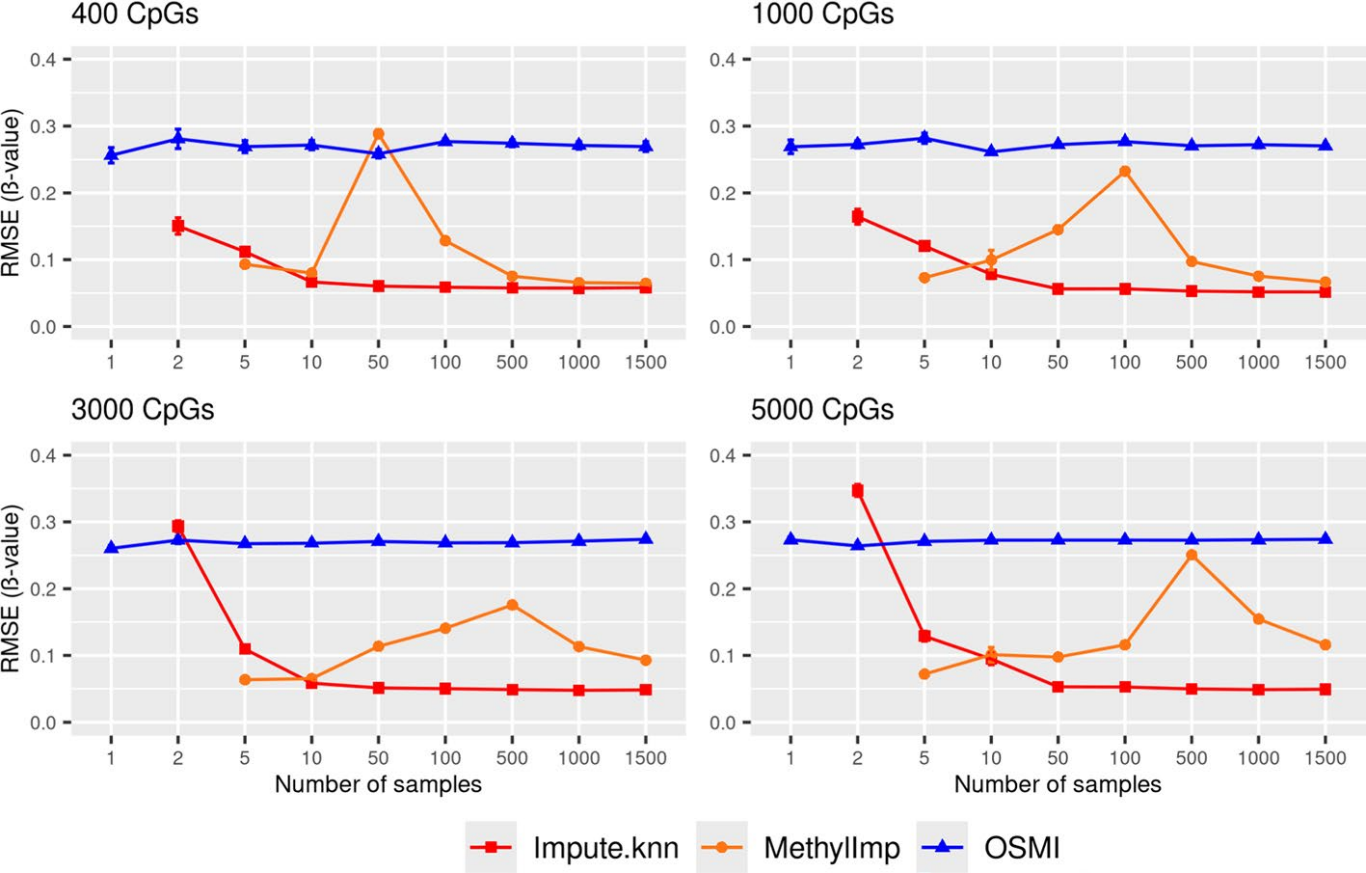
Accuracy of missing DNA-methylation value imputation with impute.knn, methyLImp, and OSMI for varying number of samples and CpG sites in the blood data set. Markers represent mean RMSEs and error bars represent standard errors of the mean. RMSEs are reported in β -value units (range: 0–1)

As expected, the RMSE of OSMI tends to be constant across varying scenarios. In contrast, methyLImp and impute.knn benefit from both more available CpG sites and samples. Compared to methyLImp and impute.knn, OSMI generally underperforms. The imputation accuracy in terms of the RMSE is summarized in Fig. 4 for all three methods with different numbers of CpG sites and samples. Note that methyLImp and impute.knn rely on the availability of multiple samples and cannot be applied in a single-sample case. For this reason, we do not show their mean RMSE values (Fig. 4). Moreover, even in the case of two available samples, methyLImp was not successfully applicable.

A similar pattern was observed when the MAE was computed (see Fig. 1 Supplement).

**Fig. 5:**
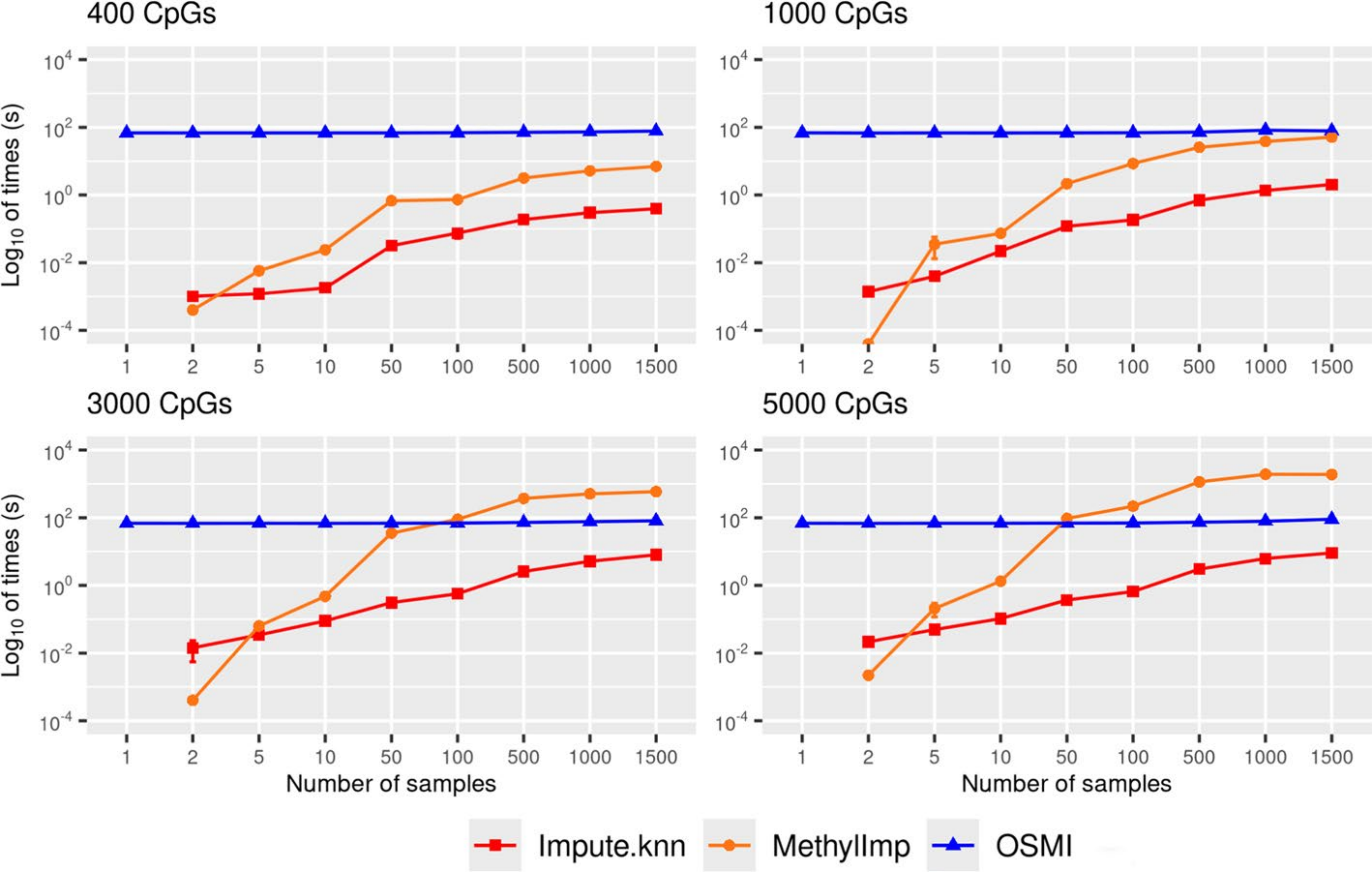
Computational time in seconds of missing DNA-methylation value imputations with impute.knn, methyLImp, and OSMI for varying number of samples and CpG sites in the blood data set. Markers represent mean times and error bars stand for standard errors of the mean. Note that the computational times are specific to our hardware (see Sect. “Performance measures”)

All three methods differ with respect to their computational time, whereas differences become more pronounced as the data sets become larger (see Fig. 5). For small data sets, less than 1000 CpG sites and 100 samples, all methods are very fast on our hardware (see 2.4). However, for larger data sets, we observed considerable differences in runtimes between methyLImp and the other two approaches.

Hereby, it is important to note that, recently Plaksienko et. al. reported on a newer version of methyLImp (called methyLImp2), which is said to be faster than the previous version for large data sets because the processes are parallelized [21]. Since we worked with reduced data sets, we could not apply it in our experiments because some of our data subsets do not provide all information necessary for methyLImp2. In detail, methyLImp2 internally splits the input data by chromosomes into subsets. Our synthetic data of data set I (see Sect. “Data set I: Comparison of imputation methods” and Fig. 2) that we used to compare the different imputation methods are drastically reduced compared to the raw data. Consequently, the synthetic data are mostly too small to ensure that each chromosome is represented by at least one CpG, meaning that at least one chromosome-related subset might remain empty. This results in runtime errors of methyLImp2 (since some of our data do not meet the requirements of methyLImp2).

### Expansion of OSMI through CpG island exploitation

To investigate the effect of a privileged nearest neighbour search within CpG islands, we compared the residuals resulting from imputations without and with consideration of CpG island structure information. To achieve this, we used data set II (see Sect. “Data set II: Impact of CpG island structure”).

**Fig. 6:**
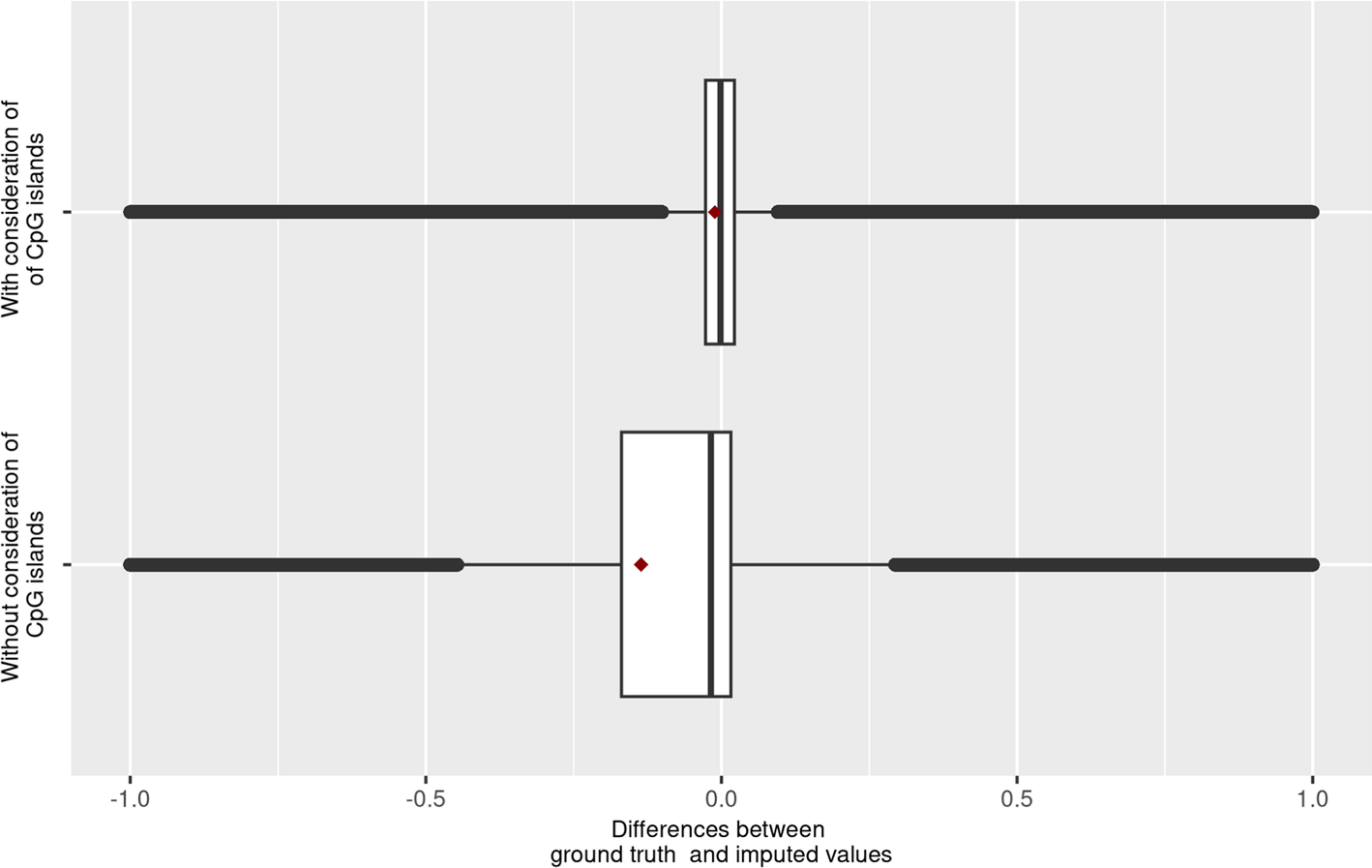
Comparison of the imputation accuracy of OSMI for CpGs located in an island, with and without prioritising the neighbour search inside the same CpG island

Using CpGs where a nearest neighbour imputation was possible within CpG islands, the imputation accuracy improved in comparison to the basic method from 0.340 to 0.1363 and from 0.200 to 0.064 in terms of RMSE and MAE respectively. Furthermore, considering the raw residuals (ground truth minus imputed values), it is noticeable that their interquartile range decreases from 0.185 to 0.049 when CpG island imputations are prioritised (see Fig. 6). Moreover, it can be seen that OSMI imputes slightly too high values on average (0.1357 increased), which is no longer observable under consideration of the CpG island structure (advanced OSMI). A statistical comparison using the Wilcoxon signed-rank test for two samples indicated a statistical significance ($p<2.2\bullet {10}^{-16}, n=\text{15.937.650}$). However, with such a large sample size, statistical significance alone can be misleading due to the high power of the test. To provide a more meaningful interpretation, we also considered rank biserial correlation and Cohen’s d as effect size measures. Both measures suggest a moderate effect with values of −0.459 and −0.399 respectively.

However, based on the original data set in Sect. “Data”, the island-based imputation expansion can only be applied to about 31% of CpGs because only 150.000 of 485.000 CpGs could be assigned to a CpG island, of which 1.000 were excluded since they were the only CpG in their corresponding island. For the remaining 69%, a replacement with the classical OSMI must be used instead. Finally, this moderate proportion reduces the advantage of the island-based imputations when applied to full data sets of current generations of microarray chips.

### Impact of the number of CpG sites on the imputation accuracy

**Fig. 7:**
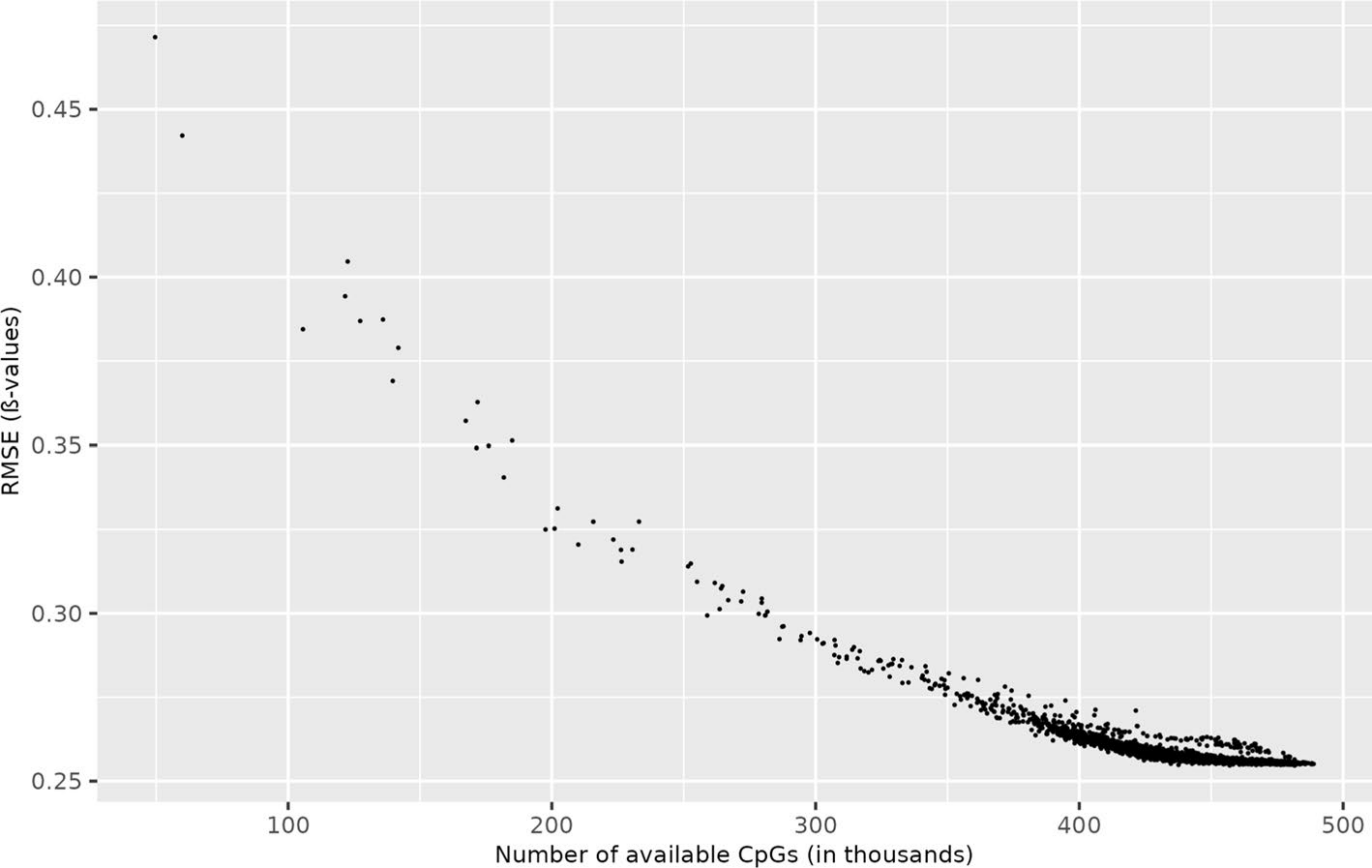
Imputation accuracy of OSMI depending on the number of available CpGs in data set III (see Sect. “Data set III: Impact of the density of a methylation array”)

We investigated the influence of the available CpG sites of one sample on the imputation accuracy of OSMI in more detail. For this purpose, we used data set III (see Sect. “Data set III: Impact of the density of a methylation array”). The special characteristic of this data set is that not all individual samples contain the same number of complete methylation values, but the number of CpGs varies depending on the original proportion of missing values per sample. The imputation accuracy across all samples based on RMSE for varying numbers of available CpG sites is shown in Fig. 7. As expected, the imputation quality tends to improve with a larger number of CpG sites. In perspective, it suggests an improved imputation accuracy as a function of CpG density.

### Influence of imputation methods on DNA-methylation clocks

Finally, we examined the impact of OSMI and other imputation methods considered in this work on the quality of DNA-methylation clocks. We mainly focus on DNA-methylation clocks as these are a popular application that may depend on the completeness of methylation measurements [3]. To illustrate this, we use Horvath’s clock as an example [19, 20].

The following results are based on the original blood data set restricted to samples, where the chronological age was available (data set IV: 485,512 Cps and 1,872 samples, see Sect. “Data set IV: Imputation effects on DNA-methylation clocks”).

Both OSMI and impute.knn could be applied to the maximum size data set with our computational resources. Whereas methyLImp could not be applied. Thus, we also used a minimum size data set, which was restricted to CpGs necessary for Horvath's Clock (353 Cps and 1872 samples). Jointly, we cover a best- and worst-case data scenario. Finally, impute.knn was applied to the 353 and 485 k CpGs data set (subsequently abbreviated as impute.knn353, impute.knn485k), methyLImp to the 353 CpGs data set only (methyLImp) and OSMI to the 485 k CpGs data set only because OSMI always uses the maximum available CpG set of a microarray chip of a single sample for the next neighbour search.

Notice that advanced OSMI was not considered in this case because the estimates of the biological age based on OSMI and advanced OSMI are strongly correlated. This is because island-based imputations are rarely used (see Sect. “Expansion of OSMI through CpG island exploitation”), and thus OSMI and advanced OSMI frequently yield identical imputations.

To compare the influence of imputation methods on DNA-methylation clocks, we set up a structural equation model (SEM) using the lavaan R package [22]. SEMs enable the evaluation of so called latent constructs/variables, i.e. variables that cannot be measured directly, in our case biological age (age). In contrast, the SEM takes measured (manifest) variables as input (i.e. methyLImp, impute.knn353, impute.knn485k, OSMI).

**Fig. 8:**
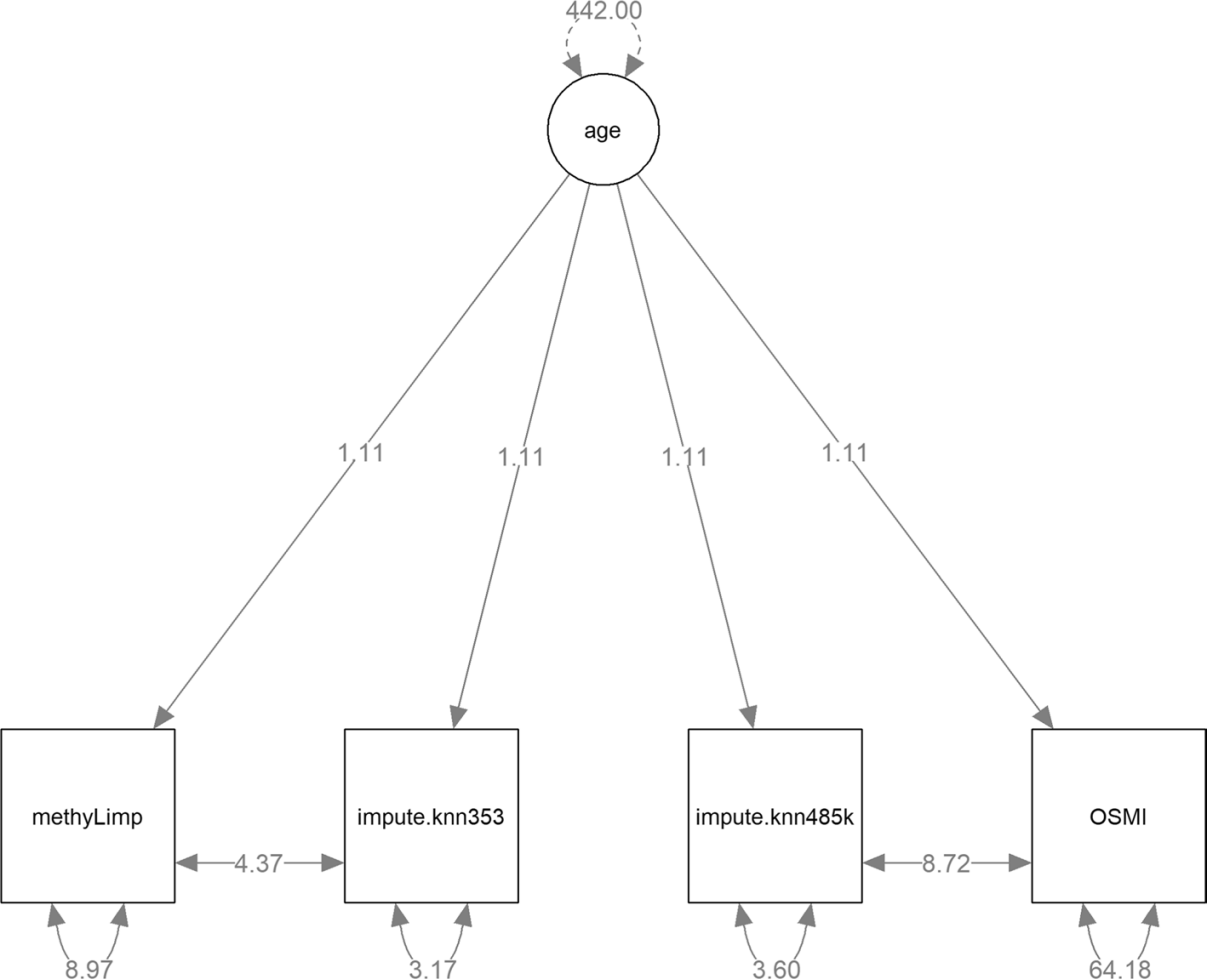
SEM for the four age prediction methods. Solid two-headed arrows represent residual (co-) variances of the measurement variables

Including all four methods in a common SEM for biological age enables the assessment of each method's reliability. Each measurement variable represents biological age as a combination of the true (latent, unobservable) biological age and measurement error. The SEM estimates residual variance, which quantifies how much of each method's variance stems from measurement error, with the remainder attributable to the actual biological age being predicted. Therefore, higher residual variance indicates lower reliability of a method in measuring biological age, as it contains greater measurement errors on average. That is, the considerably higher residual variance of 64.18 (Fig. 8) related to OSMI indicates a lower reliability of this method in comparison to the other methods. Globally, this reflects the underperformance of OSMI compared to the two comparison methods when several samples are available.

Figure 8 displays the SEM and the estimated parameters. Note that we present a restricted model in which the loadings that were nearly identical in the unrestricted model have be constrained to the same estimated value (both models had an equally good fit). To make the model identifiable, we fixed the variance of the latent age variable to the variance of the chronological age in the sample. In this case, this is equal to 422. The residuals of *impute.knn353* were allowed to co-vary with those of *methyLimp* because both methods use the 353 CpGs data set. Analogously, the residuals of *impute.knn485k* and *OSMI* may co-vary because both methods use the 485 k CpGs data set.

Based on the SEM in Fig. 8, one can compare the residual variances of OSMI with all other methods. Overall, estimates of biological age by OSMI were more volatile compared to the estimates from the other imputation methods (Table 2), as indicated by the large positive residual variance differences.

**Table 2:** Comparison of residual variances of OSMI with the other methods. Higher residual variances indicate a less reliable estimation of the latent biological age

Model contrast	Estimate of residual variance difference	95% confidence interval
*OSMI–impute. knn353*	61.01	54.33*–*67.69
*OSMI–impute.knn485k*	60.57	56.39*–*64.76
*OSMI–methyLimp*	55.20	48.49*–*61.91

## Discussion

DNA-methylation data measured by microarray or sequencing technologies frequently suffer from missing values. The way in which the missing data are processed may have a major impact on downstream analyses. Available approaches to impute missing DNA-methylation values rely on measured values from other samples to estimate such missing values assuming similarities of DNA-methylation in individuals from the same population.

However, due to more personalized analyses, interest in methods that exclusively rely on the same individual is growing. Here we present OSMI, a method that relies on the observation of co-methylation between nearby CpG sites on the same chromosome and strand within a single sample.

According to the construction of the method, the imputation accuracy of OSMI is independent of the number of available samples, which implies that the imputation precision does not improve if several samples are available. In contrast, methods such as impute.knn or methyLImp benefit from an increasing number of available samples.

Additionally, OSMI is directly dependent on the density of CpG sites. While our work used publically available 485 K DNA-methylation arrays, it became apparent that there are often no neighbouring CpGs within a distance of 50 base pairs. This distance was suggested as suitable nucleotide distance to exploit nearby correlation structures [11]. We observed that the proportion of imputations based on nearest neighbours less than or equal to 50 base pairs away was on average 5.0%. Overall, the proportion of imputations based on chromosome or methylome-wide averages amounted to 5.3%. Note that unavailable CpG positions also contributed to this observation.

Turning to strengths, OSMI is an approach driven by biological considerations related to the clustering of CpG sites in CpG island structures. It enables imputation within a single sample and does not borrow information on DNA-methylation at the same CpG site from other individuals.

Both the memory requirements and the computational time that is needed are low. Moreover, we could show that the approach becomes more precise the more densely CpGs sites are available, and when prioritising the neighbour search inside CpG islands. In this respect, a performance improvement may go hand in hand with an enhancement in DNA-sequencing technologies and increasing microarray chip sizes. Advancements on single-cell sequencing technologies suggest a potential relevance of imputing missing DNA-methylation values to improve the quality and resolution of methylation data, particularly from limited or single samples [23].

While sample based imputation methods deliver superior precision in the presence of data of several subjects, OSMI stands alone in its ability to process individual cases, making it indispensable for rare genetic disorders, personalized tumour profiling, rare diseases, or case studies where only one sample is available [23–26]. Depending on the research question OSMI may either be applied alone, e.g. for the case of individualized DNA-methylation clock predictions, or in combination with methods that benefit from information in the other samples. In the latter case, OSMI may contribute as sensitivity analyses to explore the robustness of the findings.

## Conclusion

OSMI is an imputation method for DNA-methylation data that can be applied to single-sample applications. It may become a more widely applicable imputation tool as personalized medicine develops and starts to produce more single samples for DNA-methylation analyses.

## Supplementary Information

Additional file 1.

## Data Availability

The method is freely available as R function (https://zenodo.org/records/10984523). This work is based on data from the EWAS data hub which is a subdomain of the open source platform EWAS https://ngdc.cncb.ac.cn/ewas/datahub/download, version v1.20210621. CpG start positions, information about chromosomes and strands as well as information on CpG islands were obtained from: http://hgdownload.cse.ucsc.edu/goldenpath/hg38/database (file: cpgIslandExt.txt.gz) and https://bioconductor.org/packages/IlluminaHumanMethylation450kanno.ilmn12.hg19 (file: IlluminaHumanMethylation450kanno.ilmn12.hg19_0.6.1.tar.gz).

## Abbreviations

CpG: Cytosine-phosphate-guanine
MAE: Mean absolute error
OSMI: One-sample methyl imputation
RMSE: Root mean square error
SEM: Structural equation model
