# Supplementary material for: One-sample missing DNA-methylation value imputation
Source: BMC Bioinformatics. 2025 May 31;26:143. doi: 10.1186/s12859-025-06154-9 (PMC12126866; doi:10.1186/s12859-025-06154-9)
Supplement: Supplementary file 1 — Additional file 1. [file 12859_2025_6154_MOESM1_ESM.docx]

**Supplementary material**


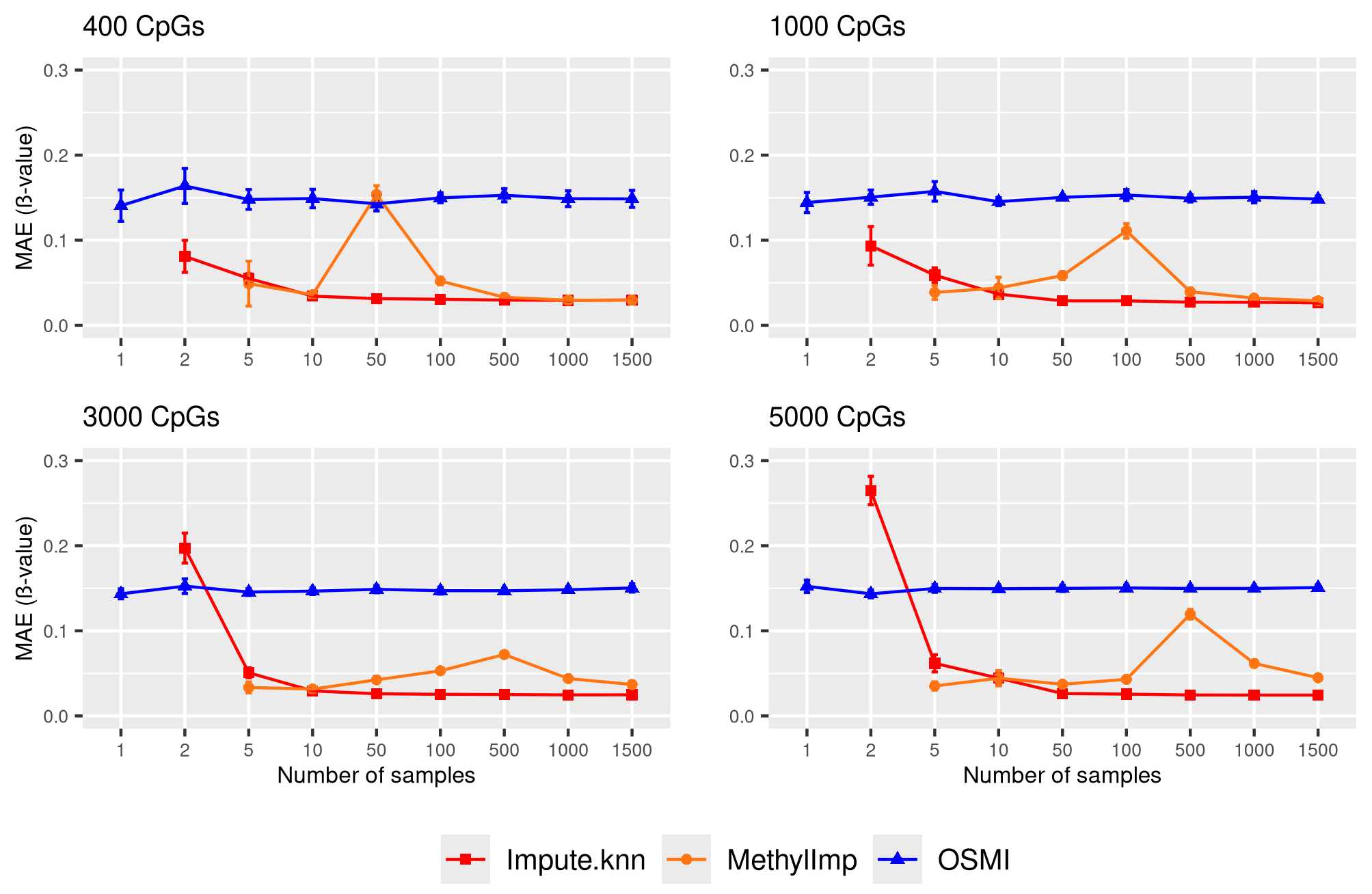


**Figure 1 Suppl:** Accuracy of missing DNA-methylation value imputation with impute.knn, methyLImp, and OSMI for varying number of samples and CpG sites in the blood data set. Markers represent mean MAEs and error bars represent standard errors of the mean. MAEs are reported in β-value units (range: 0-1).
